# Multiple Routes of Pesticide Exposure for Honey Bees Living Near Agricultural Fields

**DOI:** 10.1371/journal.pone.0029268

**Published:** 2012-01-03

**Authors:** Christian H. Krupke, Greg J. Hunt, Brian D. Eitzer, Gladys Andino, Krispn Given

**Affiliations:** 1 Department of Entomology, Purdue University, West Lafayette, Indiana, United States of America; 2 Department of Analytical Chemistry, The Connecticut Agricultural Experiment Station, New Haven, Connecticut, United States of America; Ghent University, Belgium

## Abstract

Populations of honey bees and other pollinators have declined worldwide in recent years. A variety of stressors have been implicated as potential causes, including agricultural pesticides. Neonicotinoid insecticides, which are widely used and highly toxic to honey bees, have been found in previous analyses of honey bee pollen and comb material. However, the routes of exposure have remained largely undefined. We used LC/MS-MS to analyze samples of honey bees, pollen stored in the hive and several potential exposure routes associated with plantings of neonicotinoid treated maize. Our results demonstrate that bees are exposed to these compounds and several other agricultural pesticides in several ways throughout the foraging period. During spring, extremely high levels of clothianidin and thiamethoxam were found in planter exhaust material produced during the planting of treated maize seed. We also found neonicotinoids in the soil of each field we sampled, including unplanted fields. Plants visited by foraging bees (dandelions) growing near these fields were found to contain neonicotinoids as well. This indicates deposition of neonicotinoids on the flowers, uptake by the root system, or both. Dead bees collected near hive entrances during the spring sampling period were found to contain clothianidin as well, although whether exposure was oral (consuming pollen) or by contact (soil/planter dust) is unclear. We also detected the insecticide clothianidin in pollen collected by bees and stored in the hive. When maize plants in our field reached anthesis, maize pollen from treated seed was found to contain clothianidin and other pesticides; and honey bees in our study readily collected maize pollen. These findings clarify some of the mechanisms by which honey bees may be exposed to agricultural pesticides throughout the growing season. These results have implications for a wide range of large-scale annual cropping systems that utilize neonicotinoid seed treatments.

## Introduction

Pollinator health is receiving increased attention as both managed pollinators (i.e. honey bees) and native pollinator populations decline worldwide [1]–[3]. Several causal mechanisms (including viral pathogens, parasitic mites and pesticides) have been proposed and investigated as contributing causes [4]. Pesticide exposure has received significant attention and recently-published analyses of pollen from managed bees located near agricultural environments demonstrated that many agricultural chemicals (including insecticides, miticides, fungicides and herbicides) are detectable in honey bee wax and pollen samples [5], [6]. Of the many compounds detected, the neo-nicotinoid group has arguably received the most attention. These compounds act as nicotinic acetylcholine receptor agonists in insects, causing persistent excitation of these receptors and eventually death [7]. As a group, neonicotinoids possess several key attributes that have facilitated heavy adoption in both agricultural and urban environments, including low vertebrate toxicity [8] and the ability to be translocated by plants. Neonicotinoids are also persistent, offering the potential for a large window of activity. The half-lives of these compounds in aerobic soil conditions can vary widely, but are best measured in months (148–1,155 days for clothianidin) [8]. Among the largest single uses of these compounds is application to maize seed; production of maize for food, feed and ethanol production represents the largest single use of arable land in North America. Maize planting reached unprecedented levels in the US in 2010 (35.7 million hectares [9], and is expected to increase. Virtually all if the maize seed planted in North America (the lone exception being organic production = 0.2% of total acreage [9]) is coated with neonicotinoid insecticides. There are 2 major compounds used: clothianidin and thiamethoxam; the latter is metabolized to clothianidin in the insect. The current application rates for these compounds range from 0.25 to 1.25 mg/kernel. These compounds are highly toxic to honey bees: a single kernel contains several orders of magnitude of active ingredient more than the published LD50 values for honey bees (defined as the amount of material that will kill 50% of exposed individuals), which ranges from 22–44 ng/bee for clothianidin (contact toxicity) [8], [10]. Maize seeds are typically planted at a rate ca. 12,500 kernels/hectare, making it essential that any potential routes for honey bee exposure to these compounds be quantified as thoroughly as possible. This study was initiated in response to reports of bee kills at Indiana apiaries in spring of 2010. These reports coincided with the peak period of maize planting in the area [11]. Results of analyses of these bees and pollen from the hives revealed that both clothianidin and thiamethoxam were present on dead bees and in pollen collected from a single hive. These compounds were also present in dead bees from other hives but not in bees from hives that did not show mortality. Also found was atrazine, a herbicide that is commonly used in maize production and is relatively non-toxic to honey bees [12].

These preliminary results prompted additional experiments to determine how honey bees may be gaining exposure to clothianidin and other pesticides commonly applied to either maize seed or to plants later in the season. We collected samples from a variety of potential exposure routes near agricultural fields and analyzed them to determine whether pesticides were present. We sampled soils, pollen (both collected by honey bees and directly from plants), dandelion flowers, and dead and healthy bees. We also examined waste products produced during the planting of treated seed. Sowing of maize seed in North America is accomplished using tractor-drawn planters that employ a forced air/vacuum system and a perforated disc to pick up individual seeds and drop them into the planting furrow at the selected spacing. Because maize kernels are treated with neonicotinoids and other compounds (usually fungicides) they do not flow readily and may stick to one another, causing uneven plant spacing. To remedy this, talc (a common mineral composed of hydrated magnesium silicate) is typically added to seed boxes to reduce friction and stickiness and ensure smooth flow of seed during planting. Much of the talc is exhausted during planting, either down with the seed or behind the planter and into the air using an exhaust fan. We sampled waste talc following planting to determine whether this material was contaminated with pesticides abraded from treated seeds. This waste is a mixture of the talc that has been in contact with treated maize kernels and minute pieces of the seeds themselves.

## Results

Soil collected from areas near our test site revealed that neonicotinoid insecticide residues were present in all samples tested (Table 1), with clothianidin occurring in each field sampled. Herbicide residues were also found in these samples. Sampling of the waste talc from planting activities revealed that extremely high concentrations of clothianidin were found in talc exposed to treated seed (Table 2). Fungicides applied to the seed were also found. Analysis of talc used to plant untreated seed contained low quantities of the same pesticides, this is likely due to contamination and reflects the difficulties associated with thorough cleaning of equipment between plantings. Direct sampling of anthers revealed that many of the same compounds were present in maize grown from treated seed, albeit in far lower concentrations (Table 3). Collection of pollen from traps in the field demonstrated that thiamethoxam was present in 3 of 20 samples, while pollen containing clothianidin was present in 10 of 20 samples (Table 4). Fungicides were also frequently detected: azoxystrobin and propiconazole were found in all pollen samples, while trifloxystrobin was found in 12 of the 20 samples analyzed. Maize pollen was frequently collected by foraging honey bees while it was available: maize pollen comprised over 50% of the pollen collected by bees, by volume, in 10 of 20 samples.

**Table 1:** Pesticide concentrations found in soil samples taken from production fields surrounding study area, all concentrations shown are expressed as parts per billion.^1^^,^ 2

FIELD HISTORY	THIAMETHOXAM LOD = 1.0	CLOTHIANIDIN LOD = 2.0	IMIDACLOPRID LOD = 1.0	METOLACHLOR LOD = 2.0	ATRAZINE LOD = 0.5
MAIZE-MAIZE^*^	ND	**6.3**	**2.9**	**5.9**	**52.0**
SOY-SOY	ND	**9.6**	**7.3**	**11.1**	**7.8**
MAIZE-SOY	ND	**4.9**	**ND**	**6.1**	**8.5**
SOY-MAIZE	ND	**2.1**	**ND**	**ND**	**22**

1 ND  =  Not detected.

2 *  =  Experimental field where hives were placed in 2010.

**Table 2:** Pesticide concentrations found in talc samples removed from planter following planting with treated and untreated maize seed, all concentrations shown are expressed as parts per million.^1^^,^ 2

SEED TYPE	THIAMETHOXAM LOD = 0.02	CLOTHIANIDIN LOD = 0.04	METALAXYL LOD = 0.04	TRIFLOXYSTROBIN LOD = 0.04
TALC ONLY	**ND**	**ND**	**ND**	**ND**
COMMERCIALLY TREATED MAIZE SEED 1	**735**	**3400**	**116**	**66**
COMMERCIALLY TREATED MAIZE SEED 2**	**68**	**10000**	**92**	**3.8**
COMMERCIALLY TREATED MAIZE SEED 3	**13240**	**4900**	**263**	**503**
COMMERCIALLY TREATED MAIZE SEED 4	**70**	**15030**	**131**	**4.4**
COMMERCIALLY TREATED MAIZE SEED 5	**588**	**11413**	**116**	**189**
UNTREATED MAIZE SEED***	**12**	**12**	**4**	**4**

1 ND  =  Not detected.

2 **, ***  =  Talc samples taken following planting of treated and untreated sections of experimental fields, respectively, in 2010.

**Table 3:** Pesticide concentrations found in pollen removed from maize anthers at anthesis. Samples were taken from the experimental field where hives were placed. All concentrations shown are expressed as parts per billion.^1^

	THIAMETHOXAM LOD = 0.2	CLOTHIANIDIN LOD = 0.5	METALAXYL LOD = 0.5	TRIFLOXYSTROBIN LOD = 0.5
TREATED MAIZE SEED 2	**1.7**	**3.9**	**3.1**	**1.7**
UNTREATED MAIZE SEED	**ND**	**0.3**	**4.0**	**5.5**

1 ND = Not detected.

**Table 4:** Pesticide concentrations found in pollen samples removed from returning foragers of hives placed adjacent to maize fields at planting, all concentrations shown are expressed as parts per billion.^1^

HIVE NUMBER	DATE	% MAIZE POLLEN	THIAMETHOXAM LOD = 0.5	CLOTHIANIDIN LOD = 1.0	TRIFLOXYSTROBIN LOD = 0.4	AZOXYSTROBIN LOD = 0.5	PROPICONAZOLE LOD = 2.0
HIVE #5: Untreated	**2 d pre-planting**	**82.7**	**ND**	**ND**	**1.6**	**22**	**9.3**
	**1 d pre-planting**	**78.5**	**ND**	**20.1**	**3.9**	**23**	**12.5**
	**At planting**	**54.7**	**ND**	**ND**	**17.9**	**9.6**	**15.0**
	**1 d post planting**	**16.4**	**ND**	**ND**	**18.0**	**9.2**	**9.6**
	**2 d post planting**	**56.5**	**ND**	**ND**	**1.6**	**82**	**41.0**
HIVE #7: Untreated	**2 d pre-planting**	**58.8**	**ND**	**1.1**	**ND**	**1.4**	**7.3**
	**1 d pre-planting**	**73.4**	**ND**	**13.1**	**ND**	**17.9**	**10.9**
	**At planting**	**47.9**	**ND**	**ND**	**0.4**	**18.9**	**5.6**
	**1 d post planting**	**8.8**	**ND**	**6.7**	**0.9**	**12.9**	**14.3**
	**2 d post planting**	**52.9**	**ND**	**3.4**	**0.4**	**26.9**	**15.8**
HIVE #6: Treated	**2 d pre-planting**	**69.1**	**ND**	**ND**	**0.5**	**12.4**	**5.6**
	**1 d pre-planting**	**76.3**	**ND**	**ND**	**ND**	**5.4**	**7.6**
	**At planting**	**59.5**	**ND**	**ND**	**1.8**	**7.1**	**4.8**
	**1 d post planting**	**18.2**	**ND**	**ND**	**ND**	**66**	**23.8**
	**2 d post planting**	**18.7**	**ND**	**ND**	**1.2**	**7.5**	**6.5**
HIVE #8: Treated	**2 d pre-planting**	**43.0**	**7.4**	**88**	**9.8**	**30.5**	**9.2**
	**1 d pre-planting**	**24.0**	**2.3**	**25**	**3.3**	**11.3**	**9.5**
	**At planting**	**35.4**	**1.2**	**10**	**2.6**	**6.7**	**6.6**
	**1 d post planting**	**2.6**	**ND**	**12**	**2.1**	**7.5**	**9.1**
	**2 d post planting**	**13.5**	**ND**	**3.9**	**2.6**	**4.3**	**3.2**

1 ND = Not detected.

The samples collected in 2011 revealed some similar trends (Table 5); clothianidin was found on all the dead and dying bees we sampled, while the apparently healthy bees we sampled from the same locations did not contain detectable levels of clothianidin. Atrazine and metolachlor were also found, providing further evidence that these bees were foraging near agricultural fields; as these herbicides are commonly applied prior to or during maize planting. When we sampled the contents of wax combs removed from two hives at the same apiary, we found both clothianidin and thiamethoxam in pollen removed from both hives. Nectar did not contain either compound. The miticide coumaphos was found at low levels in each nectar and pollen sample as well. Both soil and dandelion flowers obtained from the fields closest to the affected apiary (soybeans in 2010, unplanted when sampled in 2011) contained clothianidin (Table 6), therefore clothianidin in/on the dandelions could have resulted from translocation from the soil to the flower, from surface contamination of the flowers from dust, or a combination of these two mechanisms. Dandelion flowers growing far from agricultural areas served as controls; no neonicotinoids were detected.

**Table 5:** Pesticide concentrations found in/near apiary colonies during planting period in 2011, all concentrations shown are expressed as parts per billion.^1^

SAMPLE TYPE	Sample wt. (g)	THIAMETHOXAM LOD = 0.5	CLOTHIANIDIN LOD = 1.0	METOLACHLOR LOD = 2.0	ATRAZINE LOD = 0.5	AZOXYSTROBIN LOD = 0.5	COUMAPHOS LOD = 1.0
Dead/dying bees	2.96	**ND**	**6.9**	**3.0**	**6.5**	**ND**	**ND**
Dead/dying bees	2.47	**ND**	**10.8**	**1.7**	**3.9**	**ND**	**ND**
Dead/dying bees	1.32	**ND**	**3.8**	**5.5**	**9.5**	**ND**	**ND**
Dead/dying bees	2.57	**ND**	**4.9**	**0.8**	**4.6**	**ND**	**ND**
Dead/dying bees	1.62	**ND**	**13.3**	**1.1**	**3.9**	**ND**	**ND**
Healthy bees	0.59	**ND**	**ND**	**ND**	**5.9**	**ND**	**ND**
Nectar hive 1 (healthy)	5.78	**ND**	**ND**	**ND**	**0.5**	**0.6**	**1.1**
Nectar hive 2 (sick)	5.72	**ND**	**ND**	**ND**	**ND**	**0.3**	**4.7**
Pollen hive 1 (healthy, 2 samples)	5.05	**6.2±4.9**	**2.9±1.3**	**28.5±3.5**	**16±1.4**	**28.5±3.5**	**1.3±0.4**
Pollen hive 2 (sick, 2 samples)	5.08	**20.4±2.7**	**10.7±2.3**	**81.5±0.7**	**36.5±3.5**	**0.8±0.3**	**2.7±0.3**

When two aliquots of the same sample were analyzed the results are expressed as ± the standard deviation of the two analyses.

1 ND = Not detected.

**Table 6:** Pesticide concentrations found in unplanted fields near apiary during planting period in 2011, all concentrations shown are expressed as parts per billion.^1^

SAMPLE TYPE	Sample wt. (g)	THIAMETHOXAM LOD = 1.0	CLOTHIANIDIN LOD = 1.0	METOLACHLOR LOD = 0.5	ATRAZINE LOD = 0.2	AZOXYSTROBIN LOD = 0.2	COUMAPHOS LOD = 1.0
Soil, unplanted field 1, Soybeans 2010 (2 samples)	5.15, 5.01	**ND**	**6.0±0.3**	**1014±14**	**771±170**	**0.2±0.1**	**ND**
Soil, unplanted field 2, Soybeans 2010 (2 samples)	5.28, 5.43	**ND**	**8.9±0.1**	**8.3±0.7**	**160±15**	**26±17**	**ND**
Dandelions near maize field	2.96	**ND**	**1.4**	**49**	**677**	**ND**	**ND**
Dandelions near maize field	3.81	**1.6**	**5.9**	**64**	**1133**	**ND**	**ND**
Dandelions near maize field	4.51	**1.3**	**3.1**	**28**	**522**	**ND**	**ND**
Dandelions near maize field	4.05	**2.9**	**1.1**	**60**	**269**	**ND**	**ND**
Dandelions near maize field	3.10	**1.1**	**1.6**	**5.7**	**125**	**ND**	**ND**
Dandelions near maize field	3.44	**ND**	**9.4**	**295**	**1004**	**ND**	**ND**
Dandelion, CAES (non-agricultural area)	3.93	**ND**	**ND**	**ND**	**0.3**	**ND**	**ND**

When two aliquots of the same sample were analyzed the results are expressed as ± the standard deviation of the two analyses.

1 ND = Not detected.

## Discussion

The results we present here more clearly define some potential intersections between foraging honey bees and some of the seed treatments used during planting of maize. These results demonstrate that honey bees living and foraging near agricultural fields are exposed to neonicotinoids and other pesticides through multiple mechanisms throughout the spring and summer. The potential for greatest exposure (and the period when mortality was noted), occurs during planting time when there is potential for exposure to extremely high concentrations of neonicotinoids in waste talc that is exhausted to the environment during and after planting. Furthermore, we show that bees living in these environments will forage for maize pollen and transport pollen containing neonicotinoids to the hive. Pollen contaminated with levels of neonicotinoids similar to those shown in our results has been known to impair pollinator health [13], [14], [15]. Although we anticipated that planter dust may cause higher pesticide concentrations in samples taken immediately after planting our plots, we found the opposite trend: Pollen collected just prior to our planting period contained the highest levels of neonicotinoids detected (and not detected) in samples from both the treated and untreated fields. This may reflect the high variability in the types of pollen being brought back to the hive. Most of the maize in our study area was shedding pollen before and during our atypically late planting period (mid-July). After sampling anthers directly and identifying maize pollen in our samples, we know that pollen originating from treated seed does contain clothianidin, although not at the levels found in some of the bee-collected pollen samples, indicating the likelihood of additional pathways or sources. The levels of clothianidin in bee-collected pollen that we found are approximately 10-fold higher than reported from experiments conducted in canola grown from clothianidin-treated seed [16].

Detection of agricultural pesticides (and neonicotinoids in particular) in hives (including honey, pollen and wax) has been documented in the past: Bee-collected pollen was found to contain the neonicotinoid imidacloprid [17] in one study, although no adverse effects upon adults or brood were found [18]. However, a more recent study found that rearing brood in comb contaminated with pesticides (including the neonicotinoids found in our study, thiamethoxam and clothianidin) led to delayed worker development [6]. A field study examining the effects upon honey bees of clothianidin-treated canola found low levels of clothianidin in both pollen and nectar (0.93ppb and 2.59 ppb, respectively), but also found no significant effects upon honey bee populations [16]. In studies in maize, guttation droplets produced by plants grown from neonicotinoid treated seed were shown to have from 10–100 mg/L of the pesticides and were found to cause paralysis and eventual death when fed to honey bees [19], while other studies have found traces of the seed treatment imidacloprid on vegetation near maize plantings and have hypothesized that sowing treated seed can cause dispersal of dust containing insecticide [20]. Further evidence of detrimental effects of planting treated maize seed was noted by researchers in Italy, who found that honey bee mortality increased on the day seeds were planted and that numbers of foragers declined in the days following planting [21]. A subsequent study demonstrated that bees that were induced to fly near a maize planter in Europe showed up to 100 ng of clothianidin/bee upon analysis. Interestingly, however, these bees did not die unless they were kept in conditions of high humidity [22].

Detection of clothianidin in pollen, both in stored pollen in cells and in pollen traps is a critical finding because clothianidin is even more toxic when administered to bees orally, with an LD50 of 2.8–3.7 ng/bee [23], [24]. Given an average weight of 80–100 mg/bee, some of our pollen sample concentrations exceed the oral LD50. This, combined with the result that our samples of dead and dying honey bees consistently demonstrated the presence of clothianidin, suggests that the levels of both clothianidin and thiamethoxam found in our sampling of stored pollen in May of 2011 may have contributed to the deaths of the bees we analyzed. However, our analytical methods do not allow us to determine what fraction of the pesticide is on the surface of bees (contact toxicity, due to drift of soil or planter exhaust) vs. inside the body (oral toxicity, due to ingestion of contaminated pollen or guttation droplets). A combination of these exposure modalities is not unlikely.

Our results also demonstrate that clothianidin is present in the surface soil of agricultural fields long after treated seed has been planted in that field. All soil samples we collected contained clothianidin, even in cases where no treated seed had been planted for 2 growing seasons. During the spring planting period, dust that arises from this soil may land on flowers frequented by bees, or possibly on the insects themselves. Of potentially greater concern are the very high levels of neonicotinoids (and fungicides) found in the talc that has been exposed to treated seed, since part of this highly mobile material is exhausted to the outside environment during planting and after planting. The large areas being planted with neonicotinoid treated seeds, combined with the high persistence of these materials and the mobility of disturbed soil and talc dust, carry potential for effects over an area that may exceed the boundaries of the production fields themselves. A key mechanism for honey bee exposure may occur during the period when maize is typically planted across much of the Midwest (mid-April through early May). At this time, the energetic requirements of honey bee colonies are increasing rapidly and pollen and nectar resources are being gathered for colony growth. Talc and soil dusts from planting are mobile and have the potential to contaminate any flowering plants that are commonly found in or near agricultural fields and are visited by honey bees, including dandelion (*Taraxacum officinale*), which has been shown to be a preferred pollen and nectar source for honey bees during this period, when floral resources are relatively limited [25].

Later in the season, when planting is largely complete, we found that honey bees will collect maize pollen that contains translocated neonicotinoids and other pesticides from seed. Translocation of neonicotinoids into pollen has previously been reported for maize grown from imidacloprid-treated seed [26], although the degree to which honey bees in our study gathered maize pollen was surprising. The finding that bee-collected pollen contained neonicotinoids is of particular concern because of the risks to newly-emerged nurse bees, which must feed upon pollen reserves in the hive immediately following emergence. Pollen is the primary source of protein for honey bees, and is fed to larvae by nurse bees in the form of royal jelly. A bee will consume 65 mg of pollen during the 10 day period it spends as a nurse bee [27], therefore a concentration of 20 ng/g (ppb) in pollen would correspond to a dose of 1.3 ng (65 mg×20 ng/g) or almost 50% of the oral LD50 of ca. 2.8 ng/bee [23]. Some of our pollen concentrations were even higher, although it is important to note that LD50 is measured as a one-time dose, while exposure through contaminated pollen would be spread out over the 10 d period and that there is likely substantial metabolic decay of the compounds during this time. Lethal levels of insecticides in pollen are an obvious concern, but sublethal levels are also worthy of study as even slight behavioral effects may impact how affected bees carry out important tasks such as brood rearing, orientation and communication.

Also potentially important are the three fungicides found in bee-collected pollen samples (trifloxystrobin and azoxystrobin and propiconazole). Azoxystrobin and trifloxystrobin are frequently used in maize seed treatments as protectants and all three of these compounds are also widely applied to maize in North America, even in the absence of disease symptoms [28]. These compounds are typically applied using aerial application during anthesis. Propiconazole has been shown to synergize toxicity of some neonicotinoids (thiacloprid and acetamiprid) to honey bees in the laboratory, although the same results have not been shown in field studies [10], [29]. Although these fungicides are not acutely toxic to honey bees [5], the fact that they are routinely applied to areas that bees will frequent (i.e. maize plants at anthesis) coupled with the difficulties and uncertainties in assessing the toxicity of pesticide mixtures [30], indicate that they should be considered in future work.

In evaluating our results, it is important to bear in mind that toxicity is only one variable in addressing pesticide risks to pollinators – the intersection between toxicity and exposure is key in determining how much risk is posed by a toxicant to a given organism. These components are assessed by regulators in developing a “risk cup” which combines these parameters to assess the cumulative risks of a given toxicant to an organism [31]. In the case of honey bees, the toxicity of the neonicotinoid seed treatments used for large acreage field crops has been well-established [14], [31], although when assessing the overall threat to posed to honey bee populations, calculations are complicated even further by the observation that sublethal doses of insecticides can weaken bees and increase susceptibility to key parasites or pathogens [32].

Because we found these compounds in pollen, oral LD50 is a relevant parameter in discussing toxicity to honey bees. In terms of acute toxicity (based on the oral LD50 of 2.8 ng/bee [23]), the amount of clothianidin on a single maize seed at the rate of 0.5 mg/kernel contains enough active ingredient to kill over 80,000 honey bees. However, the overall level of risk has been more difficult to quantify, as there has not been a clear mechanism whereby honey bees could be exposed to high levels of these compounds – once the treated seed is planted, opportunities for honey bee exposure to concentrations of neonicotinoids over a wide area should drop dramatically (although see [19]). Our results suggest that of the factors we quantified in this study, used talc exhausted during and after planting (the latter would occur during routine cleaning of planting equipment) stands out as potential routes for exposure that should be prioritized for further quantification and remediation. A recently published review of the risks posed by planting treated seeds in the E.U. estimates that measures taken there may reduce the dust generated during planting by 99% [33]. In North America, different planting equipment is used and there are currently no guidelines for disposal of waste talc, nor are there devices for filtering exhaust material from the vacuum planting systems. Producers may be largely unaware that this material is highly toxic to pollinators. However, given the unprecedented levels of maize production across the United States, coupled with the increasing adoption of neonicotinoid seed treatments in other annual crops covering a wide area, including soybeans (31.3 million ha), wheat (24.7 million ha), and cotton (4.4 million ha, all figures 2010 planting) [9], it is clear that this material presents a risk that is worthy of further investigation and possibly corrective action.

Our findings have implications both for honey bees located near these crops year-round, but also for migratory colonies (bees used to pollinate winter-flowering specialty crops in western North America, such as almonds and other fruit and nut crops). Many of these colonies reside in areas where treated seed is used extensively (i.e. the upper Midwestern United States) during the period from early spring through late fall. During this period, these bees forage on a variety of crops that may be planted using neonicotinoid treated seed, including maize, soybeans and canola. Although our study was confined to honey bees, these results are relevant for any pollinators that forage in or near agricultural fields, both in the crop itself or on other flowering plants (i.e. weeds) that are present in or near the field.

## Materials and Methods

### Ethics statement

No specific permits were required for the described field studies. All studies were performed at a Purdue Agricultural Center, which is comprised of agricultural land that is owned and maintained by Purdue University specifically for research trials. All products used during maize planting (seed, herbicides, fertilizer etc.) were registered for legal use and were applied in accordance with label guidelines. These field studies did not involve endangered or protected species.

We based our study design upon planting practices, which helped us to identify several risk factors: during maize planting, tractor movement and wind generate large quantities of dust (i.e. disturbed soil) that may land directly on bees and/or the flowers that they visit, making this dust one potential route of exposure for foraging bees.

### Sample preparation and chemical analysis

All samples were analyzed using a modified version of the QuECHeRs protocol [34]. The pesticides were extracted from the matrix (i.e. soil, talc or pollen) by agitation with water, acetonitrile and the salts magnesium sulfate and sodium acetate. After centrifuging, a portion of the supernatant acetonitrile layer containing the pesticides was further cleaned by the addition of solid phase dispersants (primary secondary amine, magnesium sulfate, C-18 silica). An aliquot of solvent was then concentrated and analyzed by liquid chromatography/mass spectrometry utilizing an Agilent 1200 LC interfaced to a Thermo –LTQ mass spectrometer. The MS was operated in a positive electrospray mode with a separate MS/MS scan function utilized for each pesticide monitored. This analytical technique provides both qualitative (retention time and mass spectra) and quantitative information. The combined techniques allow the various pesticides to be unambiguously identified at the parts per billion concentration level.

### Pollen identification

A portion of each pollen sample from 2010 experiments was used to count and identify the types of pollen collected by the bees. The identification of maize pollen was accomplished using comparisons to reference slides prepared from maize pollen in our study area. The other pollen types were identified using comparisons to online images (www.polleninfo.org). The four pollen types identified were maize (*Zea mays*), common dandelion (*Taraxacum officinale*), plantain (*Plantago* spp.) and goldenrod (*Solidago* spp.). To estimate the proportion of maize pollen present, a subsample of pollen suspended in water was pipetted onto a disposable hemacytometer (DRM-700 Cell-VU CBC) and the pollen grains were counted. Fresh maize pollen grains are prolate spheroidal in shape [35]. The maize pollen volume was estimated measuring the principal diameters of five individual grains from each subsample, resulting in an average volume for each maize pollen grain of 3.69×10^−4^ mm^3^.

### Field experiment design, planting and sample collection methods

Our experimental field was surrounded by fields that had been planted with maize and soybeans. We sampled soil collected from the experimental field and each of the adjacent fields. Soil samples of 500 cc were taken at four equidistant locations within each field on July 7, 2010. Because we were most interested in the content of dust that may arise from disturbed soil, we confined our sampling to the top 2–3 cm of soil. The soil samples from each field were pooled and mixed prior to analysis. A 500 cc sample of soil was taken from each pooled sample for chemical analysis. On July 8 2010, we placed 8 hives surrounding an unplanted field in an area of long-term maize and soybean planting. The field dimensions were approximately 142 m wide by 148 m long. This area was divided in half, and the hives were then arranged such that each field half had a hive in the center of each border. Hive entrances faced the field interior. Pollen traps (Model #M00682, Dadant and Sons, Inc. M. Hamilton, IL) were placed in the entrances of each hive and pollen samples collected daily both before and after the field was planted. Samples were labeled and stored at −10°C prior to analysis. Hives were selected for approximately equal populations. Each hive contained about 20,000 to 30,000 bees. This level of stocking is much lower than other apiaries that are kept closer to Purdue University, and not expected to result in excessive competition for resources. However, honey bees will forage up to several kilometers if necessary and individual hives will vary in their foraging needs and resource preferences. We chose this number of hives to give an adequate sampling of the agricultural fields in which they were placed, without the expectation that they would forage primarily in the experimental field.

On July 12 2010, we planted half of the field with commercial maize seed treated with 1.25 mg/kernel of clothianidin, adding talc to each seed box at the recommended rate (approx. 240 cc talc/75 kg of maize seed) [36]. Because untreated maize seed was not available commercially, we used maize harvested the previous year to plant the other half of the field. Talc was added to this seed at the same rate as above. Fields were planted using a 6-row John Deere 7200 MaxEmerge planter. Collection of waste talc for analysis was performed following planting by manually removing approximately 50 g of talc from the manifold of the planter vacuum system. The planter and vacuum system was exhausted thoroughly and cleaned with compressed air prior to each planting and following each collection at a location far from the experimental fields. Samples of fresh pollen from maize anthers were taken by removing the entire tassel from approximately 100 plants in the field while pollen was being shed. Samples were vigorously shaken into paper bags. The resulting mixture of pollen and anthers was spread over paper towels. Anthers and other large debris were removed so that only pollen grains remained for analysis.

### 2011 sampling

During the spring of 2011, we again received reports of dead and dying bees at a local apiary, located in a small wooded area near maize and soybean production fields in northwestern Indiana. As in 2010, these reports coincided with local planting and tillage activities. We collected bees from the entrances of several hives on May 10^th^ and 12^th^, 2011. We also collected apparently healthy returning foragers from hives at the same apiary. We removed frames containing nectar and pollen from two colonies at this location: one frame was taken from a hive with dead bees near its entrance, the second frame was removed from a nearby hive without any dead bees visible. Pollen and nectar from these frames was removed from cells for analysis, and two separate analyses of pollen samples were conducted. Finally, we collected samples of surface soil (using the methods outlined above) and dandelion flowers (multiple areas sampled, approximately 7–10 flowers were collected/sample) from maize fields within 2 km of this apiary that were being planted at the time of our bee collections.

We also collected additional waste talc samples in 2011, using commercially available neonicotinoid treated maize seed from several different manufacturers. Because our goal was to develop a representative sample from a variety of maize hybrids used in our research area, all hybrids were selected based upon agronomic suitability for local planting. Both clothianidin and thiamethoxam treated seed was used, at application rates ranging from 0.25 mg/kernel to 1.25 mg/kernel. Talc was added to each seed box at the recommended rate (approx. 240 cc talc/75 kg of maize seed) (36). Fields were planted using a 6-row John Deere 7200 MaxEmerge planter. Collection of waste talc for analysis was performed following planting by manually removing approximately 50 g of talc from the manifold of the planter vacuum system using a scoopula. The planter and vacuum system was exhausted thoroughly and cleaned with compressed air prior to each planting and following each collection.
